# Preparation of a magnetic polystyrene nanocomposite for dispersive solid-phase extraction of copper ions in environmental samples

**DOI:** 10.1038/s41598-020-60232-x

**Published:** 2020-02-24

**Authors:** Ali Mehdinia, Maede Salamat, Ali Jabbari

**Affiliations:** 1 Iranian National Institute for Oceanography and Atmospheric Science, P.O. Box: 141554781, Tehran, Iran; 2 Department of Chemistry, Faculty of Science, K. N. Toosi University of Technology, Tehran, Iran

**Keywords:** Environmental sciences, Chemistry

## Abstract

The core shell nanostructure of magnetic polystyrene (PS@Fe_3_O_4_) was prepared and its physic-chemical properties were studied FT-IR, SEM, TEM, VSM and BET + BJH. The new adsorbent was applied in the dispersive solid phase extraction technique for measuring copper ions in water, Soil and Oyster samples. Analysis is carried out using a flame atomic absorption spectrometry system. Effective parameters on extraction efficiency, such as pH of extraction solution, sorbent dosage, contact time, concentration and volume of desorption eluent and desorption time were optimized using one at a time method. N_2_ adsorption-desorption experiment resulted in high BET surface area (32.002 m^2^ g^−1^) and large pore volume (0.1794 cm^3^ g^−1^) for PS@ Fe_3_O_4_ nanocomposite. Under the optimum conditions, a calibration curve within the range of 5–40 ng mL^−1^ with an appropriate coefficient of determination (R^2^) of 0.9946 was obtained. Preconcentration factor (PF) and limit of detection (LOD) were found to be 55 and 1.6 ng mL^−1^, respectively. The repeatability and reproducibility for three replicate measurements at the concentration of 25 ng mL^−1^ were 2.5%–1.4%, respectively. The Freundlich adsorption isotherm and pseudo-second-order kinetic model were consistent to experimental data in adsorption mechanism study. The maximum adsorption capacity was 19.56 mg g^−1^ for Cu (II). Finally, the efficiency of the method was investigated for analysis of the copper in environmental samples and good relative recoveries (RR%) were obtained within the range of 99.2% to 101.2%.

## Introduction

Environmental pollutants are one of the most important global challenges that cause the destruction of natural ecosystems. The development of industry, power plants, mining industries as well as agricultural activities helps to increase environmental pollutants in different forms and concentrations^1^. Among these pollutants, heavy metals which enter the environment through industrial activities, natural deposits, plating plants, mining and alloy production^2^ are important. The presence of heavy metals has adverse effects such as high blood pressure, speech disorders and memory loss on human health^3^. High concentrations level of copper ions, as one of the environmental pollutants, cause cancer of the lungs, gastrointestinal and mucous irritation, liver and kidney problems^4^. The high concentrations of copper ions in biological systems (more than 0.15 mg L^−1^ for fish and 0.01 mg per liter for invertebrates) can be fatal. In the agricultural sector, copper has the role of regulating the growth and reproduction of plants, and optimizing the amount of this element is important because copper deficiency can have adverse effects on photosynthesis, reproduction, protein formation and auxin regulation^5^. Due to the fact that heavy metals are very stable and soluble, they can be absorbed by living organisms, so the removal of these metals is of great importance. Various methods e.g. membrane filtration, ion exchange, absorption, chemical precipitation, liquid-liquid extraction and electrochemical precipitation have been used to remove heavy metals^6^. Some of these methods have drawbacks, for instance, waste materials (e.g. iron-based silt or hydroxide) produced from other processes in chemical precipitation method, can act as new reagents. If the silt contains heavy metals, it will be regarded as dangerous waste and will be accompanied by high processing costs. In ion exchange technique, non-ionized bodies are not capable of ion exchange. The chemicals used in this method are more than other methods and it’s a costly technique. The adsorption method is very popular due to its high performance, simplicity, variety of adsorbents and low cost^7^.

In recent years, nanoparticles have attracted considerable attention as adsorbent, according to their unique physical and chemical characteristics. Some of these structures can be pointed to the small size and increased surface to volume which gives them different thermal, mechanical and electronic properties. These nanomaterials include metals, polymers, semiconductors, and carbon-based materials^8,9^. In the early 1990s, researchers described the spherical symmetric core-shell structures. Properties of these structures, in addition to their size, are also related to the nature of core and shell materials. These properties include chemical, physical, catalytic, electrical and optical properties. In simple mode, these structures are made of a material and in a complex state made of two or more different materials. The choice of these materials is highly dependent on the application. These structures are generally classified into four categories: organic-organic, inorganic-inorganic, organic-inorganic and vice versa^10^.

In recent years, polymer-based adsorbents have a good alternative to activated carbon due to their large surface area, high mechanical strength and appropriate particle size distribution. Generally, polymer-based adsorbents can adsorb most of the contaminants, including metal ions, effectively. Some polymers that were previously used for extraction in conjunction with magnetic iron nanoparticles include polyaniline^11^, polydopamine^12^, polypyrrole^13^, poly(meth-acrylic acid)^14^, poly(sodium acrylate)^15^, and poly(pyrrole-co-aniline)^16^. Polymeric adsorbents are the best option for use because of their high structural strength, surface functionalities, and harmlessness to the environment^17^.

Until now, polystyrene has been used as a coating with other compounds such as polyaniline^18^ and silica^19^. Amino-functionalized Fe_3_O_4_-polystyrene was also used for preconcentration of some drugs in urine sample^20^. In this work, polystyrene alone has been used as a coating of core-shell structure to remove of Cu^2+^ ions from Soil, River water and Mollusks. In synthesis of the adsorbent, Fe_3_O_4_ magnetic nanoparticles were as the core and the styrene is polymerized around the magnetic core as the shell.

## Methods and Materials

### Materials

Styrene (St), ferric chloride hexahydrate (FeCl_3_.6H_2_O), ferrous chloride tetrahydrate (FeCl_2_.4H_2_O), Ammonia (25%), Ethanol, Sodium dodecyl sulfate (SDS), Potassium persulfate (KPS) and Sodium bicarbonate (NaHCO_3_) were purchased for Merck Co. (Darmstadt, Germany). The Cu (II) stock solution was prepared by dissolving a quantified amount of Cu (NO_3_)_2_ into deionized water and 1 mL of nitric acid was added to the solution to increase stability.

### Preparation of magnetic nanoparticles

The usual precipitation method was used for synthesis of Fe_3_O_4_ nanoparticles. Typically, (9.4 g) FeCl_3_, 6H_2_O and (3.5 g) FeCl_2_.4H_2_O were first dissolved in (160 mL) of distilled water. The solution was ultrasonicated for (5) min using an ultrasonic bath, then was stirred for 20 min in nitrogen atmosphere, then, (20 mL) of NH_4_OH (25%, v/v) was added to the solution. The temperature rose up to 80 °C and then the reaction continued for 30 min. The Fe_3_O_4_ NPs were gathered by a magnet (1.4 T)^21^.

### Synthesis of core shell PS@Fe_3_O_4_

To put the polystyrene outer coating on the magnetic nanoparticles, (1 g) of Fe_3_O_4_ particles was dispersed in (10 mL) of ethanol under ultrasonic waves for (30) min. Then (0.5 g) of SDS, (0.24 g) of NaHCO_3_ and (0.1 g) of KSP were added to the solution with (90 mL) of deionized water. Finally, (10 mL) of styrene was added. The reaction was carried out in a 250 mL flask at 85 °C for (10) h under N_2_ atmosphere.

## Real Samples

### Preparation of soil sample

First, 1 g of soil sample was dried at a 110 °C. (10 mL) of concentrated nitric acid was added to the sample. The mixture was heated until drying. After cooling to room temperature, a second (10 mL) portion of concentrated nitric acid was added and the procedure was repeated. Then (10 mL) of concentrated hydrochloric acid was added to the beaker and the mixture was gently heated until complete drying. After cooling, the residue was dissolved in (10 mL) of 1 M HCl. Finally, the pH of solution was set and diluted to the mark with distilled water^22^.

### Preparation of an oyster sample

The soft tissues some oyster samples (*Saccostrea cucullata*), collected from coasts of the Persian Gulf, were removed and mixed. The mixed sample was freeze-dried at −50 °C (500 mm Torr). The mixture of HNO_3_:H_2_O_2_ (9:3 mL) was added to (0.25 g) of the sample and digestion was performed for 4 h at 80 °C. The final residue was cooled to room temperature and filtered^23^.

### Preparation of water sample

River water sample was extract without any treatment. The method was applied for the extraction of Cu^2+^ ions in river water sample under optimum condition. The pH of the water was first adjusted using HCl at pH = 6. Then, the prepared sample under two conditions, with adding and without adding (25 mg L^−1^) of analyte to 50 mL of water sample, was investigated.

### Material characterization

Chemical measurements were performed with a GBC atomic absorption spectrometer (932 plus, Germany). Fourier-transform infrared spectroscopy (FT-IR) study was carried out with a Spectrum Two (ABB Bomem, Caneda) at wavelengths ranging from 400–4000 cm^−1^. The specific surface areas were estimated using the Brunauer-Emmett-Teller (BET) from company Microtrac Bel Corp model BElSORP Mini. Surface morphology of the materials was investigated by Field Emission Scanning Electron Microscopy (FESEM) from company ZEISS (Germany) model Sigma VP. A Zeiss-EM10C-100 KV transmission electron microscope was used for the transmission electron microscopy (TEM) analysis.

### Adsorption and desorption experiments

**Figure 1:**
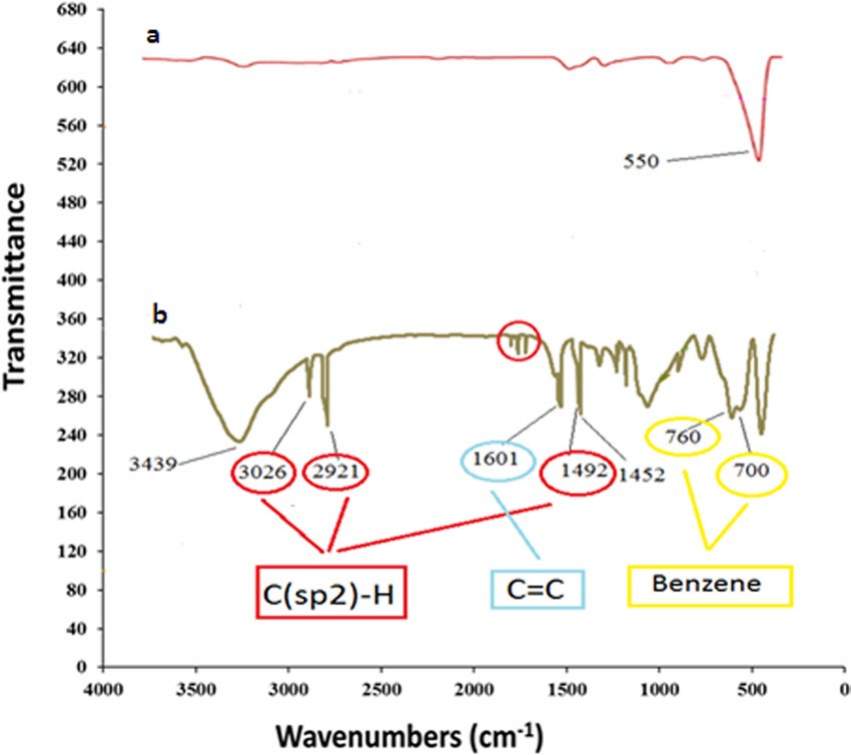
FTIR spectra (**a**) Fe_3_O_4_, (**b**) PS@Fe_3_O_4_.

To accomplish each extraction process, 50 mL of standard copper solution (5 mg L^−1^) was used. In each of the experiments, the amount of adsorbent, extraction time, and pH were calculated and applied according to a one-time variable (OVAT) optimization experiments. The graphs for each of the optimized parameters are shown in the Fig. 1S. The technique used to extract metal ions is a dispersive solid-phase extraction technique.

In each experiment, 30 mg of powder adsorbent were mixed in 50 mL standard copper solution (pH = 6) at a concentration of 5 mg L^−1^ and mixed by a magnetic stirrer for 15 min. At this stage, the adsorbent was collected by magnetic separation from the solution and injected into the flame atomic absorption to measure. Then, the residual concentrations of Cu (II) were measured. The re-adsorption test was performed using 0.5 mL nitric acid 1 molar solution to recover metal adsorbed metal ions. (Concentration, volume and type of acid are obtained using optimization experiments Fig. 1S). For this purpose, after adsorption process and collecting the adsorbent from the solution, 0.5 mL of nitric acid of 1 M was added to the adsorbent and mixed, then the solvent was removed using a magnet and injected into the flame atomic absorption. This test was performed to determine the adsorbent capability to release adsorbed metal ions.

## Results and Discussion

### Characterization study

The FT-IR spectra of Fe_3_O_4_ Fig. 1(a) and PS@Fe_3_O_4_ Fig.(1b) showed the characteristic absorption band of Fe-O around 550 cm^−1^. After the polymerization of styrene on the surface of Fe_3_O_4_, as expected, the spectrum of PS@Fe_3_O_4_ shows absorption peaks around, 2921, 3026 cm^−1^ and 1492 cm^−1^, which can be ascribed to stretching vibrations and bending vibration in polystyrene (C (sp^2^)-H). The absorbance peaks at 1601 cm^−1^ of C=C of polystyrene stretching of PS@Fe_3_O_4_ and 700–760 cm^−1^ confirmed the monosubstituted benzene.

**Figure 2:**
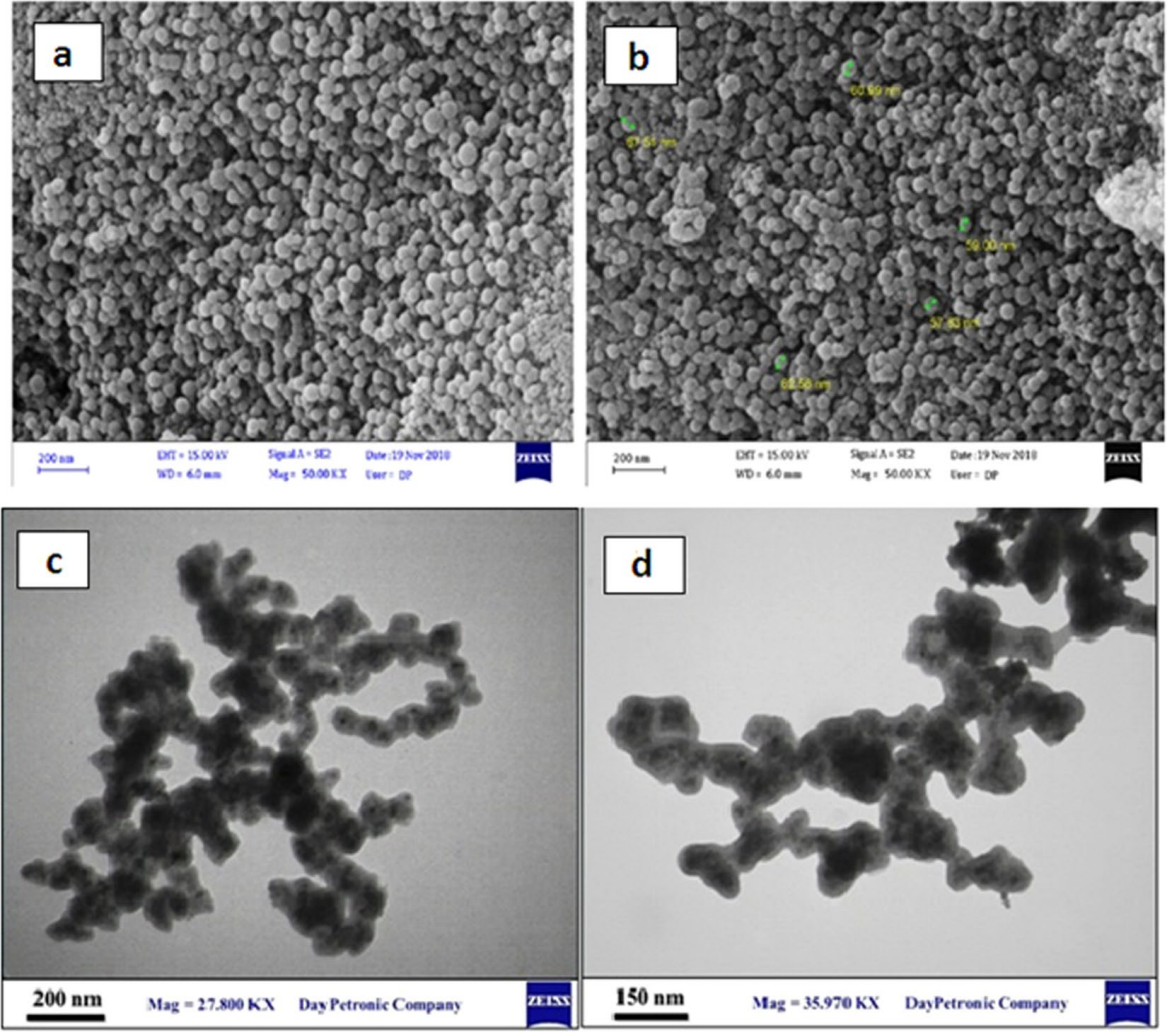
(**a,b**) SEM image of PS@Fe_3_O_4_, (**c,d**) TEM image of PS@Fe_3_O_4_.

SEM micrograph of the PS@Fe_3_O_4_ particles has been shown in Fig. 2(a,b). It can be seen that the particles have spherical shape and their sizes are around 57–67 nm. The SEM image shows that the microspheres are very uniform in both size and shape. The TEM image of magnetite particles shows the nearly spherical shape for them. Figure 2(c,d) shows that the dark magnetite particles are well wrapped with a gray PS shell.

Surface area of adsorb-synthesized materials was obtained by BET method using nitrogen adsorption. In this process, the sample was placed under vacuum at a certain temperature and time to be discharged all its pores and then it is ready to absorb nitrogen. The area was 32.002 m^2^ g^−1^ Table 1S, indicating that the PS@Fe_2_O_3_ nanorods presents a higher surface area than the pure polystyrene synthesized at the same day.

The magnetic properties of the PS@Fe_3_O_4_ were studied by VSM as shown in Fig. 2S. The saturation moments obtained from the hysteresis loop were found to be about (45 emu g^−1^) for both Fe_3_O_4_ and PS@Fe_3_O_4_. As shown in the diagram, the addition of a non-magnetic coating to the structure of magnetic material has not diminished the magnetic properties of Fe_3_O_4_. Therefore, it is expected that PS@Fe_3_O_4_ have a good respond to magnetic fields and the separation will simply happen.

### Adsorption mechanism

Investigating the mechanism of adsorption by the core-shell nanostructures depends on the outer coating of these nanoparticles. Polystyrene was studied as an outer coating in this nanostructure. Polystyrene is chemically a long chain hydrocarbon. Styrene is an aromatic molecule; aromatics are very stable compounds, so the change in the structure of polystyrene cannot be easily accomplished. To investigate the adsorption process by nanoparticles, several factors such as effective surface area are important. The mechanism of adsorption is dominated by physical process involves the electrostatic interaction between the ion in the solution and solid adsorbent, which is usually associated with low adsorption heat^24^. Ohsawa *et al*.^25^, was studied Zeta potential and surface charge density of polystyrene-latex and found that there are 2000–4000 negative charges on the its surface at pH = 7.34.

Another proposed mechanism to explain the mechanism of adsorption of copper ions can be related to the interaction of π electrons of PS and vacant orbital in Cu^2+^. In other words, cations can be act as dopant in the structure of polystyrene^26^.

### Study adsorption isotherms

The equilibrium adsorption capacity (q_e_) of the adsorbent was studied and increased up to concentrations of 30 mg L^−1^ for Cu (II). The experimental maximum adsorption capacity was 19.56 mg g^−1^ for this adsorbent according to Fig. 3S. Three important isotherms (Langmuir, Freundlich and Temkin) were studied to describe the experimental data. The initial Cu (II) concentration varied from 2 to 30 mg L^−1^. The following equation was used to represent Langmuir expression:

1 $$1/{q}_{e}=1/{q}_{m}+1/(b{q}_{m}{C}_{e})$$

where C_e_ is the equilibrium concentration of analyte (mg L^−1^), q_e_ is the amount (mg g^−1^) of adsorbed analyte at equilibrium time, and q_m_ and b are Langmuir constants related to maximum adsorption capacity (mg g^−1^) and energy of adsorption related to the heat of adsorption (L mg^−1^), respectively.

The Freundlich equation is commonly presented as:

2 $$\log \,{q}_{e}=\,\log \,{K}_{F}+(1/n)\log \,{C}_{e}$$

where K_F_ (mg g^−1^) is the Freundlich constant indicating the relative adsorption capacity of the adsorbent and n is the Freundlich equation exponent and is the parameter characterizing quasi-Gaussian energetic heterogeneity of the adsorption surface^27^.

The Temkin model is given by the following equation:

3 $${{\rm{Q}}}_{{\rm{e}}}={{\rm{K}}}_{{\rm{t}}}\,\mathrm{ln}\,{{\rm{C}}}_{{\rm{e}}}+{{\rm{K}}}_{{\rm{t}}}\,\mathrm{ln}\,{\rm{f}}$$

where K_t_ is Temkin constant belongs to the sorption heat (J mol^−1^) and f is Temkin isotherm constant.

The fitting results, obtained from the adsorption of Cu (II) on PS@Fe_3_O_4_, were listed in Table 1 and Fig. 3S. Cu (II) adsorption isotherm models could be determined based on the correlation coefficient (R^2^) in Table 1. The R^2^ values were 0.997, 0.973 and 0.658 for Freundlich, Langmuir and Temkin models, respectively. The Freundlich isotherm showed the best fitting result and it describes that the adsorption is non-ideal and reversible and also it is not restricted to monolayer formation.

**Table 1:** Kinetics and isotherm models parameters for Cu^2+^ adsorption on PS@Fe_3_O_4_.

Pseudo-first order models						Pseudo-second order models		
K_1_	q_m_	R^2^				K_2_	q_e_	R^2^
(min^−1^)	(mg g^−1^)					(g mg^−1^ min^−1^)	(mg g^−1^)	
0.0334	0.233	0.693				0.333	6.18	0.999
**Langmuir**			**Freundlich**			**Temkin**		
**q**_**m**_	**b**	**R**^**2**^	**n**	**K**_**f**_	**R**^**2**^	**f**	**K**_**t**_	**R**^**2**^
(mg g^−1^)	(L mg^−1^)			(mg g^−1^)			(J mol^−1^)	
16.52	1.933	0.973	5.15	10.45	0.997	24.3	3.588	0.658

### Study adsorption kinetics

Study of adsorption kinetics gives important information about pollutant removal in industrial process. For kinetic studies, adsorption of Cu (II) by PS@Fe_3_O_4_ was performed under the adsorption time of 5, 10, 15, 20, 25, 30 and 35 min. Here, two kinetic models, based on Eqs. (4) and (5), respectively, were used for the kinetic studies: the pseudo-first-order reaction model and the pseudo-second-order adsorption model.

4 $$\mathrm{Ln}({Q}_{e}-{Q}_{t})=\,\mathrm{ln}\,{Q}_{e,cal}-{k}_{1}t$$

5 $$t/{Q}_{t}=1/{k}_{2}{Q}_{e.cal}^{2}+t/{Q}_{e,cal}$$

where *Q*_*e*_ (mg g^−1^) and *Q*_*t*_ (mg g^−1^) are the amounts of adsorbate at equilibrium and time *t* (min), respectively; *Q*_*e*, *cal*_ (mg g^−1^) is the calculated adsorption capacity; *t* is the contact time (min); k_1_ the rate constant of pseudo-first order sorption (min^−1^) and K_2_ (g mg^−1^min^−1^) is the rate constant of the pseudo-second-order adsorption^28^. The effect of the contact time on the sorption of Cu (II) onto PS@Fe_3_O_4_ was investigated in a kinetics study of the sorption process Fig. 4S, the kinetics parameters are summarized in Table 1. It was found that the kinetics data could be best fitted into the pseudo-second-order rate model with R^2^ value higher than 0.999 for 5 mg L^−1^ of initial Cu (II) concentrations. It means that the rate of adsorption/desorption process (as a chemisorption) controls the overall sorption kinetics^29^.

### Interfering ions on Cu (II) adsorption

Different ions that are often in real samples simultaneously with copper, such as chloride (*Cl*^−^), sulfite ($S{O}_{3}^{2-}$), carbonate ($C{O}_{3}^{2-}$), chlorate ($Cl{o}_{4}^{-}$), cadmium (*Cd*^2+^), nickel (*Ni*^+^), cobalt (*Co*^2+^), magnesium (*Mg*^2+^), bromine (*Br*^−^), zinc ($Z{n}^{+}$), sodium ($N{a}^{+}$), lead ($P{b}^{2+}$), barium $(B{a}^{2+})$ and manganese ($M{n}^{2+}$) in real sample have this potential to compete with copper ions on the adsorption process. Thus, the competitive adsorption experiments were performed and corresponding results were illustrated in Table 2S. The result showed that the ions such as $C{d}^{2+}$, $\,N{i}^{+}$, $\,C{o}^{2+}$, $\,M{g}^{2+}$, $\,B{a}^{2+}$, $B{r}^{-}$, $\,Cl{o}_{4}^{-}$, $S{O}_{3}^{2-}$, $\,C{O}_{3}^{2-}$ and *Cl*^−^ to the concentration levels of 1000 times, $Z{n}^{+}$ and $N{a}^{+}$ to the concentration levels of 500 times and $P{b}^{2+}$ and $\,M{n}^{2+}$ to the concentration levels of 250 times that of the concentration of analyte almost exhibited no noticeable effect on the removal efficiency of Cu(II). Recovery achieved for each ion was 95% to 100%, indicating no disturbance. The reason for the investigation of some of the ions at a lower concentration level is the pH limitation, because these ions are precipitated in pH = 6 at higher concentrations.

The copper removal efficiency at each stage was calculated using the Eq. (6):

6 $$RE \% =\frac{{C}_{0}-{C}_{t}}{{C}_{0}}\,.\,100$$

where *C*_0_ and *C*_t_ (mg L^−1^) are the Cu (II) concentrations at initial time and time *t* (min), respectively^30^.

The following equation was used to calculate the adsorption capacity:

7 $${q}_{e}=\frac{V({C}_{0}-{C}_{e})}{m}$$

where q_e_ is the equilibrium adsorption capacity (mg g^−1^); C_0_ and C_e_ are the initial and equilibrium concentration of Cu^2+^ (mg L^−1^), respectively; V and m are the volume of solution (mL) and adsorbent dosage (g), respectively^31^.

### Analytical performance evaluation

The limit of detection (LOD) and limit of quantification (LOQ) of the present work were calculated under optimal conditions analyzed by FAAS. LOD and LOQ were defined as LOD = 3 SD/m and LOQ = 10 SD/m, where m is the slope of the calibration curve were found to be 1.6 μg L^−1^ and 5 μg L^−1^, respectively. The precision, (%RSD) using the slope of the calibration graph in terms of repeatability (data from three independent standard preparations, intra-day and inter-day %RSD), was 2.5% and 1.4%, respectively. Preconcentration factor (PF), calculated as the ratio of the slope of the extracted calibration curve to the slope of the direct calibration curve, was 55. The calibration graph obtained by using the PS@ Fe_3_O_4_ for Cu (II) was linear in the range of 5–40 ng mL^−1^ with the A = 0.0047×+0.1306 (R^2^ = 0.9946) calibration equation (The calibration curve is shown in the Fig. 5S).

### Analysis of real samples

To illustrate the applicability and reliability of the method, three environmental samples (River water, Soil and an Oyster sample) were used. For this purpose, each sample is prepared according to the method of preparation described. Then, a milliliter of the sample solution was added to 40 mL of deionized water, the pH of the samples was adjusted to pH = 6 using NaOH or HCl solution. Then the prepared samples under two conditions, with addition and without adding (25 mg L^−1^) of analyte in volume 50 mL was investigated. Table 2 shows the results of the analysis of real samples. According to the results, the recovery was in the range of 99.2–101.2%. The capability of the SPE system in the determination of copper ions was evaluated and satisfactory accuracy was obtained.

**Table 2:** Results of blank and spiked recoveries of copper in real environmental samples.

Sample	Cadded (ng mL^−1^)	Cfound+SD (ng mL^−1^)	Recovery%
Soil	0	14.1	—
	25	40.3	104 ±0 0.7
River water	0	7	—
	25	31.8	99.2 ±0 1
Oyster	0	5.2	—
	25	20.1	101.2 ±0 0.1

The proposed adsorbents were compared with other adsorbents provided for the extraction of copper ions. The results are shown in Table 3. The results indicate that the adsorbent has acceptable adsorption capacity and preconcentration factor compared to other adsorbents.

**Table 3:** Comparative data from some recent studies on copper adsorption.

Adsorbents	Adsorption Capacity (mg g^−1^)	Preconcentration Factor	Reference
Amberlite XAD-2 with PAN^a^	6.850	50	^32^
MWCNT_S_-D2EHPA^b^	4.90	25	^33^
Fe_3_O_4_ Polydopamine	28	150	^12^
Fe_3_O_4_/HAP/GQD_S_^c^	23.98	39.2	^34^
Bamboo fiber	6.0	33	^35^
PS@Fe_3_O_4_	19.56	55	This work

^a^1-(2-pyridylazo)−2-naphthol.

^b^di-(2-ethyl hexyl phosphoric acid).

^c^hydroxyapatite/graphene quantum dots.

## Conclusion

In this study, a new adsorbent (PS@Fe_3_O_4_) with core-shell structure was synthesized to extract copper ions in aqueous samples. This adsorbent was first used in the process of extraction of solid phase to remove copper ions. The synthesized adsorbent capability to adsorb copper ions in the presence of other ions, such as ($C{l}^{-}$), ($S{O}_{3}^{2-}$), ($C{O}_{3}^{2-}$), ($Cl{o}_{4}^{-}$), ($C{d}^{2+}$), ($N{i}^{+}$), ($C{o}^{2+}$), ($M{g}^{2+}$), ($B{r}^{-}$), ($Z{n}^{+}$), ($N{a}^{+}$), ($P{b}^{2+}$), ($B{a}^{2+}$) and ($M{n}^{2+}$), has been investigated and no disturbance has been observed, which shows that our adsorbant adsorbs the analyte in the presence of other ions efficiently. This adsorbent showed a significant uptake rate, more than 99.2% for real samples. The Cu (II) removal data were well fitted to the pseudo second-order kinetic model and the Freundlich adsorption model revealing that the adsorption of Cu (II) onto the PS@Fe_2_O_3_ was a reversible and not restricted to the formation of monolayer. Surface area measurements showed that the adsorbent has a high surface area 32.002 cm^2^ g^−1^ which is considered as an advantage. PS@Fe_3_O_4_ showed the maximum adsorption capacity of 19.56 mg g^−1^ for Cu (II). Due to the results and advantages expressed, absorbent PS@Fe_2_O_3_, as one of the good absorbents with high ability has been introduced to remove copper ions from aqueous solutions.

## Supporting Information

Supporting Information.
