# Supplementary material for: Preparation of a magnetic polystyrene nanocomposite for dispersive solid-phase extraction of copper ions in environmental samples
Source: Sci Rep. 2020 Feb 24;10:3279. doi: 10.1038/s41598-020-60232-x (PMC7039917; doi:10.1038/s41598-020-60232-x)
Supplement: Supplementary file 1 — Supporting Information. [file 41598_2020_60232_MOESM1_ESM.pdf]

Supplementary Information

Preparation of a magnetic polystyrene nanocomposite for dispersive solid-phase extraction of copper ions in environmental water samples aqueous

Ali Mehdinia,[*] [a], Maede Salamat [b], Ali Jabbari [b]

Iranian National Institute for Oceanography and Atmospheric Science, P.O.Box: 141554781, Tehran, Iran

[b] Department of Chemistry, Faculty of Science, K. N. Toosi University of Technology, Tehran, Iran

[*] Corresponding author: Tel: +98 21 66944873; Fax: +98 66944869.
E-mail address: a.mehdinia@inio.ac.ir (A. Mehdinia)

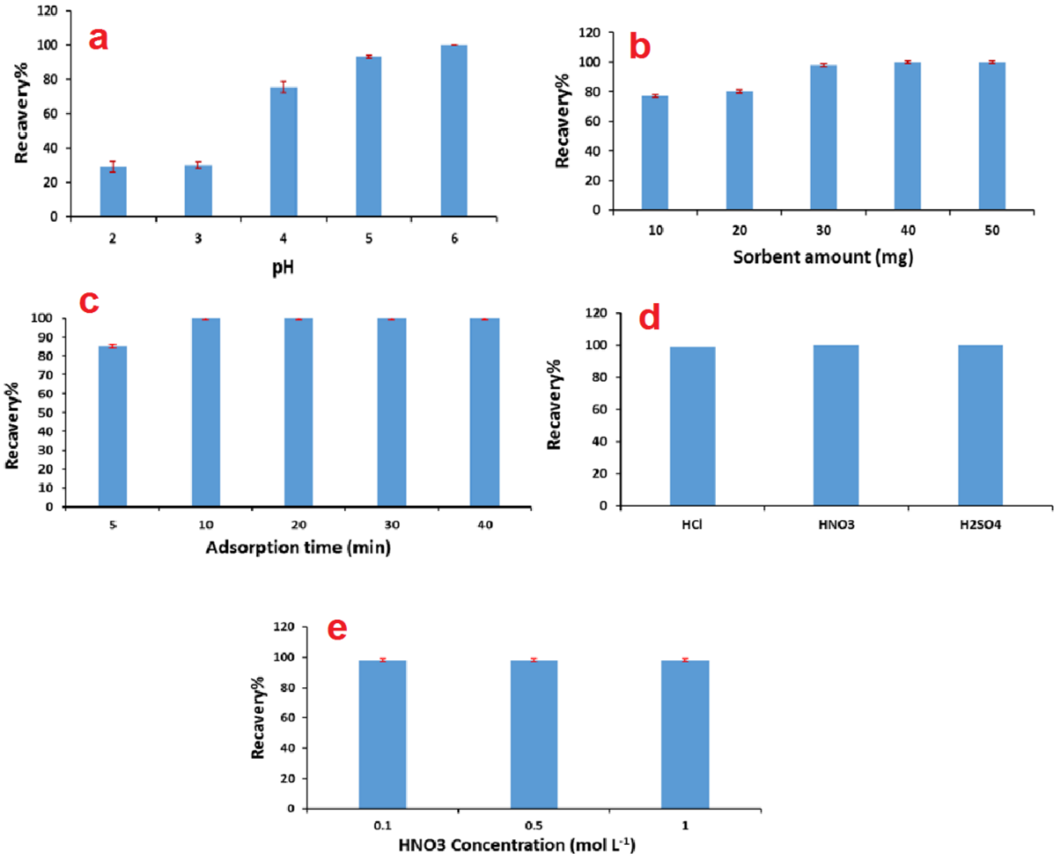

Figure 1S. Optimizations of **(a)** Effect of pH on extraction of Cu^{2+} (II), **(b)** The effect of the amount of sorbent, **(c)** The effect of adsorption time, **(d)** Effect of desorption solvent type, **(e)** Effect of concentration. (Condition: 50 mL)

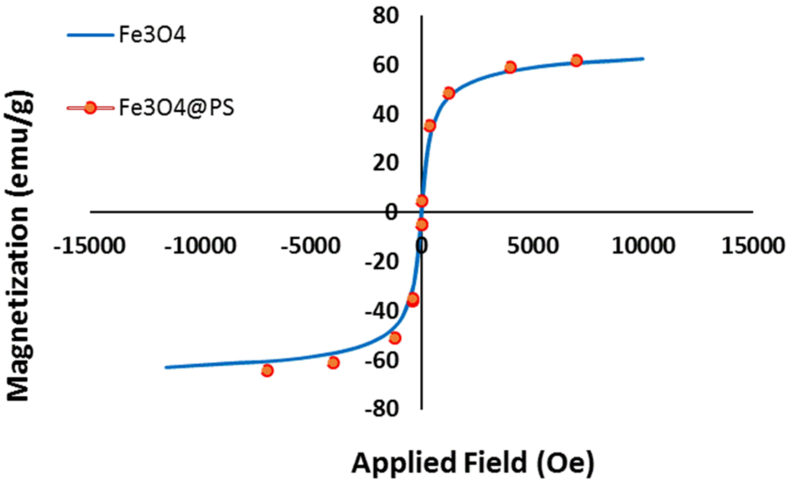

Fig 2S. VSM magnetization curves of MNPs PS@Fe_3O_4 (The insets digital images show that NPs can be easily separated from water under an external magnetic field).

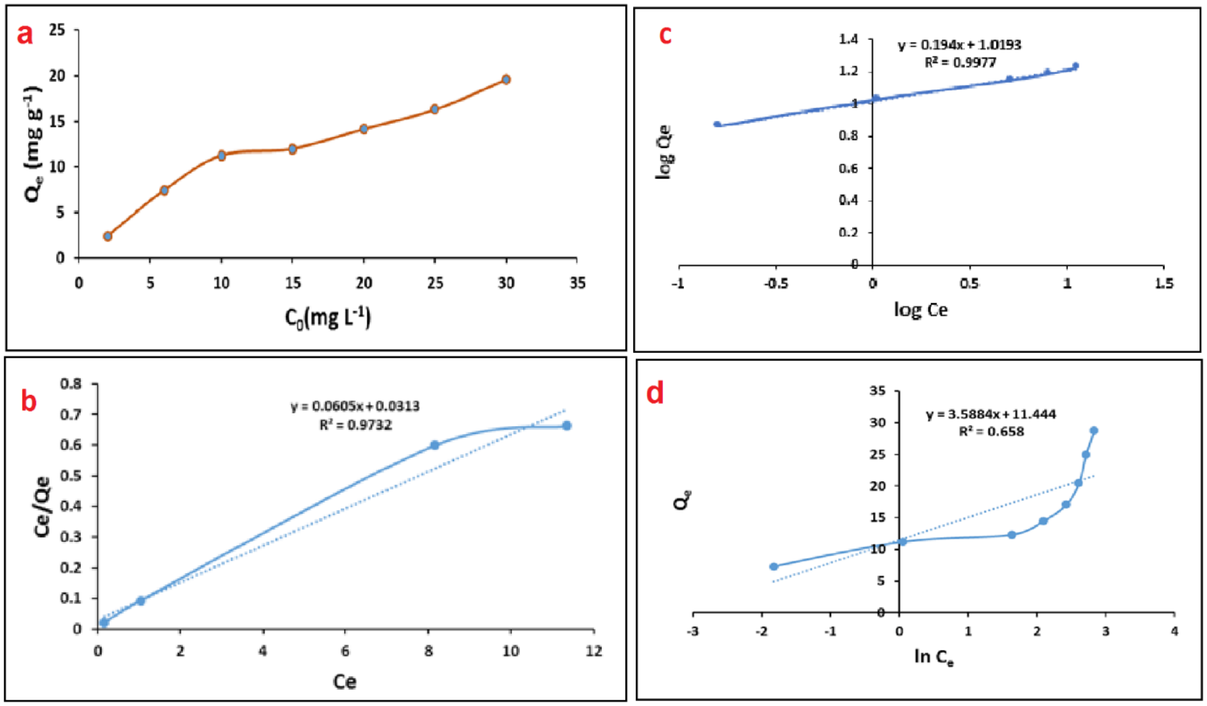

Figure 3S. Effect of initial concentration and isotherm study of Cu^{2+} on PS@Fe_3O_4 adsorbent **(a)** The plot of Q_e (mg g^{-1}) versus initial concentration of Cu^{2+} (mg L^{-1}) at temperature of 25 °C and pH of 6, curves are isotherm models, **(b)** Langmuir,**(c)** Freundlich and **(d)**Temkin for PS@Fe_3O_4.

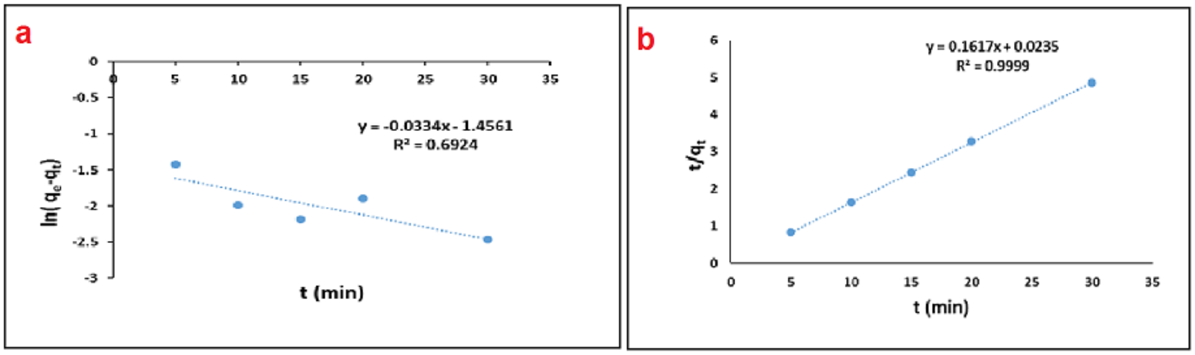

Figure 4S. Kinetic study of **(a)** Pseudo first-order and **(b)** pseudo second-order kinetic models of Cu (II) adsorption onto PS@Fe_3O_4 material (pH=6, C_0=5 mg/L).

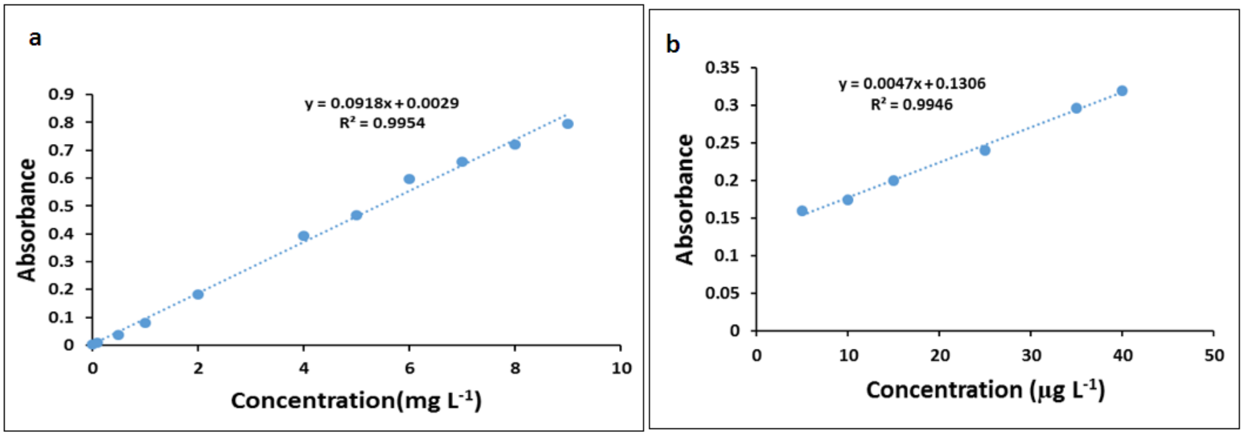

Figure 5S. (a) Direct calibration curve, **(b)** Extraction calibration curve

Table 1S. Surface area and pore volumes (BET+BJH) for PS@Fe_3O_4

	**Core shell**
Number of adsorption data	33
Number of desorption data	22
a_s, BET	32.002 m^2 g^{-1}
Total pore volume	0.1794 cm^3 g^{-1}
Mean pore diameter	22.428 nm
V_p	0.1762 cm^3 g^{-1}
r_p,peak(Area)	1.22 nm
a_p	26.802 m^2 g^{-1}

Table 2S. Effect of coexisting anions on Cu^{2+} removal by PS@Fe_3O_4

**Interfering ions**	**Ratio** $\frac{interfering}{analyte}$	**Recovery%**
Cd^{2+}	1000	96.45±0.3
Ni^{+}	1000	97.83±0.5
Co^{2+}	1000	98.40±0.6
Mg^{2+}	1000	99.31±0.3
Ba^{2+}	1000	100±0.3
Br^{-}	1000	99.52±1
Clo_4^{-}	1000	100±0.3
SO_3^{2-}	1000	97.6±0.4
CO_3^{2}	1000	100.12±0.2
Cl^{-}	1000	100.04±0.9
Zn^{+}	500	95.64±0.4
Na^{+}	500	99.6±0.6
Pb^{2+}	250	99.9±0.3
Mn^{2+}	250	95.9±0.7
